# Di-μ-thio­semicarbazide-κ^4^ *S*:*S*-bis­[chlori­dobis(triphenyl­phosphane-κ*P*)silver(I)]

**DOI:** 10.1107/S1600536812051562

**Published:** 2013-01-09

**Authors:** Yupa Wattanakanjana, Chaveng Pakawatchai, Ruthairat Nimthong

**Affiliations:** a Department of Chemistry, Faculty of Science, Prince of Songkla University, Hat Yai, Songkhla 90112, Thailand; b Department of Chemistry and Center of Excellence for Innovation in Chemistry, Faculty of Science, Prince of Songkla University, Hat Yai, Songkhla 90112, Thailand

## Abstract

The dinuclear title complex, [Ag_2_Cl_2_(CH_5_N_3_S)_2_(C_18_H_15_P)_2_], lies across an inversion center. The Ag^I^ ion exhibits a slightly distorted tetra­hedral coordination geometry formed by a P atom from a triphenyl­phosphane ligand, two metal-bridging S atoms from thio­semicabazide ligands and one chloride ion. The S atoms bridge two symmetry-related Ag^I^ ions, forming a strictly planar Ag_2_S_2_ core with an Ag⋯Ag separation of 2.7802 (7) Å. There is an intra­molecular N—H⋯Cl hydrogen bond. In the crystal, N—H⋯Cl and N—H⋯S hydrogen bonds link complex mol­ecules, forming layers parallel to (001). These layers are connected through π–π stacking inter­actions [centroid–centroid distance = 3.665 (2) Å], leading to the formation of a three-dimensional network.

## Related literature

For metal(I) complexes of phosphine ligands as precursors for the preparation of mixed-ligand complexes, see: Ferrari *et al.* (2007 ▶); Pakawatchai *et al.* (2012 ▶). For potential applications of thio­semicarbazide derivatives and their metal complexes, see: Pandeya *et al.* (1999 ▶); Wujec *et al.* (2009 ▶); Mohareb & Mohamed (2012 ▶); He *et al.* (2012 ▶). For examples of related discrete complexes, see: Wattanakanjana *et al.* (2012 ▶); Lobana *et al.* (2008 ▶).
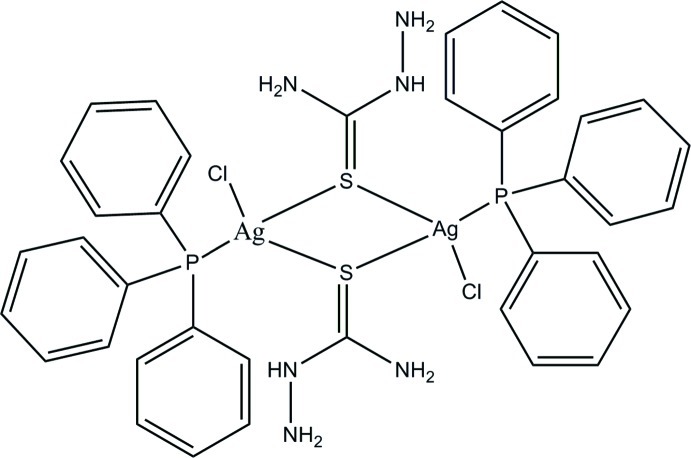


## Experimental

### 

#### Crystal data

[Ag_2_Cl_2_(CH_5_N_3_S)_2_(C_18_H_15_P)_2_]
*M* *_r_* = 993.46
Triclinic, 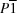

*a* = 8.7845 (4) Å
*b* = 9.4656 (4) Å
*c* = 13.7529 (6) Å
α = 109.276 (1)°
β = 98.306 (1)°
γ = 99.739 (1)°
*V* = 1038.94 (8) Å^3^
*Z* = 1
Mo *K*α radiation
μ = 1.28 mm^−1^
*T* = 293 K
0.38 × 0.30 × 0.10 mm

#### Data collection

Bruker SMART CCD diffractometer
Absorption correction: multi-scan (*SADABS*; Bruker, 2003 ▶) *T*_min_ = 0.638, *T*_max_ = 0.880
14302 measured reflections
5026 independent reflections
4627 reflections with *I* > 2σ(*I*)
*R*_int_ = 0.036

#### Refinement

*R*[*F* ^2^ > 2σ(*F* ^2^)] = 0.026
*wR*(*F* ^2^) = 0.069
*S* = 1.06
5026 reflections
251 parameters
5 restraints
H atoms treated by a mixture of independent and constrained refinement
Δρ_max_ = 0.38 e Å^−3^
Δρ_min_ = −0.55 e Å^−3^

### 

Data collection: *SMART* (Bruker, 1998 ▶); cell refinement: *SAINT* (Bruker, 2003 ▶); data reduction: *SAINT*; program(s) used to solve structure: *SHELXS97* (Sheldrick, 2008 ▶); program(s) used to refine structure: *SHELXL97* (Sheldrick, 2008 ▶); molecular graphics: *Mercury* (Macrae *et al.*, 2008 ▶); software used to prepare material for publication: *SHELXL97* and *publCIF* (Westrip, 2010 ▶).

## Supplementary Material

Crystal structure: contains datablock(s) I, global. DOI: 10.1107/S1600536812051562/lh5573sup1.cif

Structure factors: contains datablock(s) I. DOI: 10.1107/S1600536812051562/lh5573Isup2.hkl

Additional supplementary materials:  crystallographic information; 3D view; checkCIF report

## Figures and Tables

**Table 1:** Hydrogen-bond geometry (Å, °)

*D*—H⋯*A*	*D*—H	H⋯*A*	*D*⋯*A*	*D*—H⋯*A*
N3—H3*B*⋯Cl1^i^	0.87 (2)	2.67 (2)	3.535 (2)	171 (2)
N2—H2⋯S1^ii^	0.83 (2)	2.66 (2)	3.4320 (15)	155 (2)
N1—H1*B*⋯Cl1^iii^	0.85 (2)	2.63 (2)	3.4088 (16)	154 (2)
N1—H1*A*⋯Cl1	0.89 (2)	2.45 (2)	3.3239 (16)	170 (2)

Symmetry codes: (i) 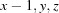; (ii) 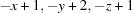; (iii) 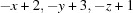.

**Fig. 1.**
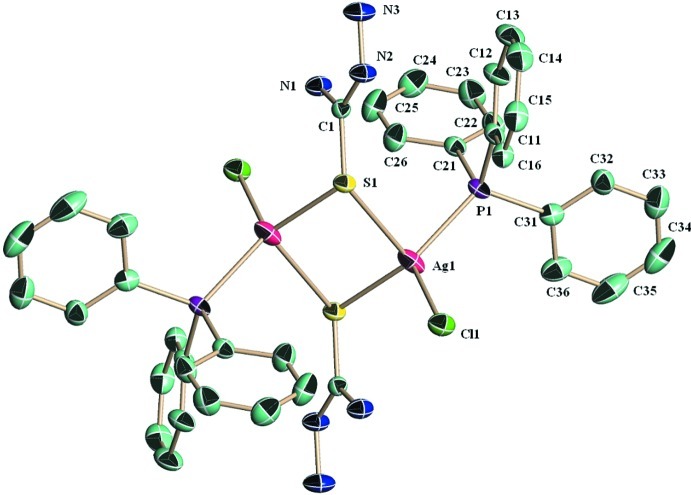
The molecular structure with displacement ellipsoids drawn at the 30% probability level. H atoms are omitted for clarity.

**Fig. 2.**
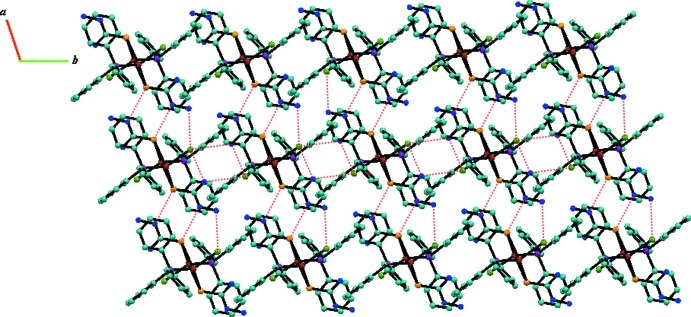
Part of the crystal structure with N—H···S and N—H···Cl hydrogen bonds shown as dashed lines.

**Fig. 3.**
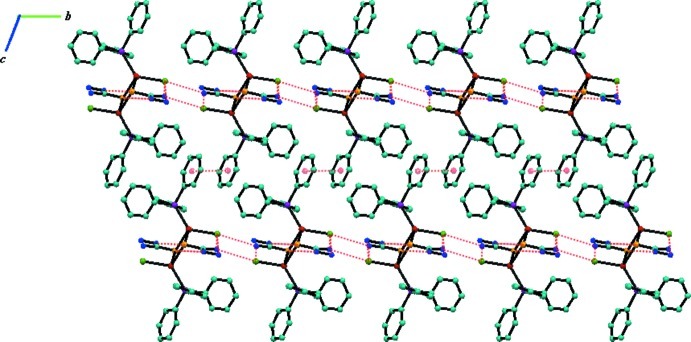
Part of the crystal structure with π–π stacking interactions shown as dashed lines.

## supplementary crystallographic information

### Comment

Metal(I) complexes of phosphine ligands have been extensively studied as precursors for preparing mixed-ligand complexes (Ferrari *et al.*, 2007; Pakawatchai *et al.*, 2012) having different geometries such as mononuclear and dinuclear. Thiosemicabazide and thiosemicarbazide derivatives, as well as their metal complexes, have recently attracted considerable attention because of their relevance in biological systems such as antitumor, antimicrobial, antibacterial and antifungal activities (Pandeya *et al.*, 1999; Wujec *et al.*, 2009; Mohareb *et al.*, 2012; He *et al.*, 2012). Herein, the crystal structure of a dinuclear silver(I) chloride complex containing triphenylphosphane and thiosemicarbazide is described.

The molecular structure of the title dinuclear compound is shown in Fig. 1. The molecule lies acroos a crystallographic inversion center which is at the center of the Ag_2_S_2_ core with a Ag···Ag separation of 2.7802 (7) Å. The bond angles around Ag^I^ ion are approximately in the range of 111.851 (15)–123.445 (15)°. Geometrical distortion from ideal angles (109.47°) can be explained by the need to accommodate the bulky triphenylphosphane groups. The P1—Ag1 bond length of 2.4225 (4) Å is slightly longer than that found in for example [Ag_2_(C_6_H_7_N_2_S)_2_(C_18_H_15_P)_2_], which is 2.4088 (6) Å (Wattanakanjana *et al.*, 2012). The bridging Ag—S bond length (Ag1—S1 = 2.5202 (4) Å) is shorter than those observed in related silver(I) complexes containing S-bridged donor ligand, due to 2.5832 (8)–2.7208 (11) Å for [Ag_2_Cl_2_(l-S-pySH)_2_(PPh_3_)_2_] and 2.6306 (4)–2.6950 (7) Å for [Ag_2_Br_2_(l-S-pySH)_2_(PPh_3_)_2_] (Lobana *et al.*, 2008). There is intramolecular N—H···Cl hydrogen bond with the geometry N1···Cl1 = 3.3239 (16) Å. In the crystal, an N1—H1*B*···Cl1 hydrogen bond connects molecules forming one dimensional chain alongs [010]. Each chain is linked through N2—H2···S1 and N3—H3*B*···Cl1 hydrogen bonds forming a layer parallel to (001) (Fig. 2). In addition, the layers are stacked *via π*···*π* stacking interactions [centroid–centroid distance = 3.665 (2) Å, centroid = C31—C36 ring] froming the three dimensional network (Fig. 3).

### Experimental

Triphenylphosphane (0.37 g, 1.41 mmol) was dissolved in 30 cm^3^ of acetonitrile at 335 K. AgCl (0.10 g, 0.70 mmol) was added and the mixture was stirred for 2.5 h. Thiosemicabazide (0.06 g, 0.66 mmol) was added and the new reaction mixture was heated under reflux for 5 h. The resulting clear solution was filtered off and left to evaporate at room temperature. The crystalline solid, which was deposited upon standing for few days, was filtered off and dried under reduced pressure.

### Refinement

All H atoms bonded to C atoms were constrained with a riding model of 0.93 Å, and *U*_iso_(H) = 1.2*U*_eq_(C). H atoms bonded to the N atoms were located in a difference Fourier map and refined isotropically, with restrained N—H distances 0.834 (16)–0.888 (16) Å with *U*_iso_(H) = 1.2*U*_eq_(N).

### Crystal data

**Table:**

[Ag_2_Cl_2_(CH_5_N_3_S)_2_(C_18_H_15_P)_2_]	*Z* = 1
*M**_r_* = 993.46	*F*(000) = 500
Triclinic, *P*1	*D*_x_ = 1.588 Mg m^−^^3^
Hall symbol: -P 1	Mo *K*α radiation, λ = 0.71073 Å
*a* = 8.7845 (4) Å	Cell parameters from 9010 reflections
*b* = 9.4656 (4) Å	θ = 2.3–28.0°
*c* = 13.7529 (6) Å	µ = 1.28 mm^−^^1^
α = 109.276 (1)°	*T* = 293 K
β = 98.306 (1)°	Block, colorless
γ = 99.739 (1)°	0.38 × 0.30 × 0.10 mm
*V* = 1038.94 (8) Å^3^	

### Data collection

**Table:**

Bruker SMART CCD diffractometer	5026 independent reflections
Radiation source: fine-focus sealed tube	4627 reflections with *I* > 2σ(*I*)
Graphite monochromator	*R*_int_ = 0.036
Full–matrix least–squares on F^2^ scans	θ_max_ = 28.0°, θ_min_ = 1.6°
Absorption correction: multi-scan (*SADABS*; Bruker, 2003)	*h* = −11→11
*T*_min_ = 0.638, *T*_max_ = 0.880	*k* = −12→12
14302 measured reflections	*l* = −18→18

### Refinement

**Table:**

Refinement on *F*^2^	Secondary atom site location: difference Fourier map
Least-squares matrix: full	Hydrogen site location: inferred from neighbouring sites
*R*[*F*^2^ > 2σ(*F*^2^)] = 0.026	H atoms treated by a mixture of independent and constrained refinement
*wR*(*F*^2^) = 0.069	*w* = 1/[σ^2^(*F*_o_^2^) + (0.0293*P*)^2^ + 0.2733*P*] where *P* = (*F*_o_^2^ + 2*F*_c_^2^)/3
*S* = 1.06	(Δ/σ)_max_ = 0.003
5026 reflections	Δρ_max_ = 0.38 e Å^−^^3^
251 parameters	Δρ_min_ = −0.55 e Å^−^^3^
5 restraints	Extinction correction: *SHELXL97* (Sheldrick, 2008), Fc^*^=kFc[1+0.001xFc^2^λ^3^/sin(2θ)]^-1/4^
Primary atom site location: structure-invariant direct methods	Extinction coefficient: 0.0693 (18)

### Special details

**Table:**

Geometry. All e.s.d.'s (except the e.s.d. in the dihedral angle between two l.s. planes) are estimated using the full covariance matrix. The cell e.s.d.'s are taken into account individually in the estimation of e.s.d.'s in distances, angles and torsion angles; correlations between e.s.d.'s in cell parameters are only used when they are defined by crystal symmetry. An approximate (isotropic) treatment of cell e.s.d.'s is used for estimating e.s.d.'s involving l.s. planes.
Refinement. Refinement of *F*^2^ against ALL reflections. The weighted *R*-factor *wR* and goodness of fit *S* are based on *F*^2^, conventional *R*-factors *R* are based on *F*, with *F* set to zero for negative *F*^2^. The threshold expression of *F*^2^ > σ(*F*^2^) is used only for calculating *R*-factors(gt) *etc*. and is not relevant to the choice of reflections for refinement. *R*-factors based on *F*^2^ are statistically about twice as large as those based on *F*, and *R*- factors based on ALL data will be even larger.

### Fractional atomic coordinates and isotropic or equivalent isotropic displacement parameters (Å^2^)

**Table:**

	*x*	*y*	*z*	*U*_iso_*/*U*_eq_	
C1	0.71808 (17)	1.20926 (18)	0.50782 (12)	0.0350 (3)	
C11	1.16762 (18)	0.7721 (2)	0.21780 (12)	0.0418 (3)	
C12	1.2004 (2)	0.6279 (3)	0.18680 (16)	0.0564 (5)	
H12	1.1194	0.5403	0.1675	0.068*	
C13	1.3557 (3)	0.6145 (3)	0.18463 (19)	0.0701 (6)	
H13	1.3781	0.5177	0.1635	0.084*	
C14	1.4747 (3)	0.7429 (4)	0.21342 (19)	0.0712 (7)	
H14	1.5778	0.7330	0.2111	0.085*	
C15	1.4436 (2)	0.8871 (3)	0.24596 (17)	0.0628 (6)	
H15	1.5255	0.9741	0.2657	0.075*	
C16	1.2904 (2)	0.9025 (2)	0.24935 (14)	0.0481 (4)	
H16	1.2695	0.9999	0.2727	0.058*	
C21	0.84762 (18)	0.61752 (19)	0.20371 (13)	0.0404 (3)	
C22	0.7696 (2)	0.5083 (2)	0.10569 (15)	0.0546 (4)	
H22	0.7794	0.5284	0.0448	0.065*	
C23	0.6776 (3)	0.3701 (3)	0.09818 (19)	0.0678 (6)	
H23	0.6257	0.2977	0.0322	0.081*	
C24	0.6619 (3)	0.3386 (3)	0.1867 (2)	0.0732 (6)	
H24	0.6023	0.2439	0.1809	0.088*	
C25	0.7351 (3)	0.4481 (3)	0.2851 (2)	0.0789 (7)	
H25	0.7228	0.4276	0.3456	0.095*	
C26	0.8265 (3)	0.5878 (3)	0.29402 (17)	0.0619 (5)	
H26	0.8737	0.6618	0.3603	0.074*	
C31	0.90601 (19)	0.8272 (2)	0.09696 (15)	0.0447 (4)	
C32	0.9697 (3)	0.7705 (2)	0.00965 (15)	0.0562 (4)	
H32	1.0525	0.7220	0.0147	0.067*	
C33	0.9124 (3)	0.7846 (3)	−0.08519 (19)	0.0758 (7)	
H33	0.9564	0.7463	−0.1433	0.091*	
C34	0.7892 (4)	0.8561 (4)	−0.0922 (3)	0.0916 (10)	
H34	0.7483	0.8642	−0.1559	0.110*	
C35	0.7272 (3)	0.9150 (4)	−0.0065 (3)	0.0931 (10)	
H35	0.6449	0.9639	−0.0121	0.112*	
C36	0.7850 (2)	0.9031 (3)	0.0888 (2)	0.0666 (6)	
H36	0.7432	0.9457	0.1472	0.080*	
N1	0.82011 (18)	1.32995 (18)	0.51108 (14)	0.0474 (3)	
N2	0.57728 (17)	1.22460 (17)	0.52697 (14)	0.0478 (3)	
N3	0.5365 (2)	1.3672 (2)	0.54628 (19)	0.0609 (5)	
P1	0.96967 (5)	0.80383 (5)	0.22142 (3)	0.03895 (10)	
S1	0.75685 (4)	1.02919 (4)	0.47995 (3)	0.03889 (10)	
Cl1	1.13857 (6)	1.28186 (5)	0.41121 (4)	0.05418 (12)	
Ag1	0.977789 (17)	1.016088 (15)	0.381354 (12)	0.05737 (8)	
H1A	0.912 (2)	1.320 (3)	0.4929 (19)	0.069*	
H1B	0.797 (3)	1.417 (2)	0.530 (2)	0.069*	
H2	0.518 (3)	1.146 (2)	0.526 (2)	0.069*	
H3A	0.528 (3)	1.404 (3)	0.6102 (14)	0.069*	
H3B	0.443 (2)	1.349 (3)	0.5070 (18)	0.069*	

### Atomic displacement parameters (Å^2^)

**Table:**

	*U*^11^	*U*^22^	*U*^33^	*U*^12^	*U*^13^	*U*^23^
C1	0.0332 (7)	0.0382 (7)	0.0346 (7)	0.0098 (6)	0.0061 (5)	0.0142 (6)
C11	0.0328 (7)	0.0549 (10)	0.0334 (7)	0.0156 (7)	0.0070 (6)	0.0082 (7)
C12	0.0476 (10)	0.0577 (11)	0.0546 (10)	0.0222 (9)	0.0088 (8)	0.0047 (9)
C13	0.0606 (13)	0.0856 (17)	0.0659 (13)	0.0444 (13)	0.0159 (10)	0.0154 (12)
C14	0.0395 (10)	0.116 (2)	0.0689 (13)	0.0367 (12)	0.0176 (9)	0.0363 (14)
C15	0.0352 (9)	0.0982 (17)	0.0597 (12)	0.0118 (10)	0.0102 (8)	0.0366 (12)
C16	0.0377 (8)	0.0630 (11)	0.0454 (9)	0.0119 (8)	0.0088 (7)	0.0218 (8)
C21	0.0336 (7)	0.0431 (8)	0.0419 (8)	0.0124 (6)	0.0080 (6)	0.0107 (7)
C22	0.0610 (11)	0.0483 (10)	0.0432 (9)	0.0011 (8)	0.0152 (8)	0.0063 (8)
C23	0.0743 (14)	0.0492 (11)	0.0610 (12)	−0.0057 (10)	0.0126 (11)	0.0063 (9)
C24	0.0709 (14)	0.0592 (13)	0.0896 (17)	0.0004 (11)	0.0118 (12)	0.0368 (13)
C25	0.0872 (17)	0.0848 (17)	0.0714 (15)	0.0060 (14)	0.0063 (13)	0.0483 (14)
C26	0.0638 (12)	0.0692 (13)	0.0483 (10)	0.0083 (10)	−0.0014 (9)	0.0249 (10)
C31	0.0343 (7)	0.0401 (8)	0.0552 (10)	0.0030 (6)	0.0044 (7)	0.0166 (7)
C32	0.0637 (12)	0.0529 (11)	0.0468 (10)	0.0099 (9)	0.0097 (8)	0.0141 (8)
C33	0.0969 (19)	0.0617 (13)	0.0547 (12)	−0.0107 (13)	0.0015 (12)	0.0234 (11)
C34	0.0875 (19)	0.0859 (19)	0.097 (2)	−0.0152 (15)	−0.0220 (16)	0.0621 (18)
C35	0.0588 (14)	0.100 (2)	0.138 (3)	0.0163 (14)	−0.0038 (16)	0.078 (2)
C36	0.0438 (10)	0.0688 (13)	0.1000 (17)	0.0183 (9)	0.0158 (11)	0.0442 (13)
N1	0.0388 (7)	0.0386 (7)	0.0699 (10)	0.0106 (6)	0.0169 (7)	0.0230 (7)
N2	0.0372 (7)	0.0396 (7)	0.0729 (10)	0.0145 (6)	0.0200 (7)	0.0226 (7)
N3	0.0468 (9)	0.0477 (9)	0.0920 (14)	0.0236 (7)	0.0164 (9)	0.0234 (9)
P1	0.03125 (19)	0.0388 (2)	0.0393 (2)	0.00988 (15)	0.00878 (15)	0.00323 (16)
S1	0.03454 (18)	0.03454 (19)	0.0490 (2)	0.01021 (14)	0.01279 (15)	0.01437 (16)
Cl1	0.0497 (2)	0.0406 (2)	0.0713 (3)	0.00658 (17)	0.0195 (2)	0.0183 (2)
Ag1	0.05604 (11)	0.04168 (10)	0.06025 (12)	0.00514 (6)	0.02583 (7)	−0.00213 (7)

### Geometric parameters (Å, º)

**Table:**

C1—N1	1.311 (2)	C25—H25	0.9300
C1—N2	1.323 (2)	C26—H26	0.9300
C1—S1	1.7236 (16)	C31—C32	1.383 (3)
C11—C12	1.383 (3)	C31—C36	1.391 (3)
C11—C16	1.391 (3)	C31—P1	1.8184 (18)
C11—P1	1.8189 (16)	C32—C33	1.386 (3)
C12—C13	1.394 (3)	C32—H32	0.9300
C12—H12	0.9300	C33—C34	1.377 (4)
C13—C14	1.364 (4)	C33—H33	0.9300
C13—H13	0.9300	C34—C35	1.360 (5)
C14—C15	1.377 (4)	C34—H34	0.9300
C14—H14	0.9300	C35—C36	1.383 (4)
C15—C16	1.384 (3)	C35—H35	0.9300
C15—H15	0.9300	C36—H36	0.9300
C16—H16	0.9300	N1—H1A	0.888 (16)
C21—C22	1.389 (2)	N1—H1B	0.851 (17)
C21—C26	1.391 (3)	N2—N3	1.406 (2)
C21—P1	1.8221 (18)	N2—H2	0.834 (16)
C22—C23	1.381 (3)	N3—H3A	0.851 (16)
C22—H22	0.9300	N3—H3B	0.870 (16)
C23—C24	1.366 (4)	P1—Ag1	2.4225 (4)
C23—H23	0.9300	S1—Ag1	2.5202 (4)
C24—C25	1.384 (4)	Cl1—Ag1	2.5378 (5)
C24—H24	0.9300	Ag1—Ag1^i^	3.3502 (4)
C25—C26	1.383 (3)		
			
N1—C1—N2	119.02 (15)	C32—C31—P1	123.22 (14)
N1—C1—S1	123.57 (12)	C36—C31—P1	118.21 (17)
N2—C1—S1	117.41 (12)	C31—C32—C33	121.2 (2)
C12—C11—C16	119.68 (16)	C31—C32—H32	119.4
C12—C11—P1	123.64 (15)	C33—C32—H32	119.4
C16—C11—P1	116.68 (13)	C34—C33—C32	119.1 (3)
C11—C12—C13	119.7 (2)	C34—C33—H33	120.5
C11—C12—H12	120.2	C32—C33—H33	120.5
C13—C12—H12	120.2	C35—C34—C33	120.4 (2)
C14—C13—C12	120.2 (2)	C35—C34—H34	119.8
C14—C13—H13	119.9	C33—C34—H34	119.8
C12—C13—H13	119.9	C34—C35—C36	120.9 (3)
C13—C14—C15	120.57 (19)	C34—C35—H35	119.6
C13—C14—H14	119.7	C36—C35—H35	119.6
C15—C14—H14	119.7	C35—C36—C31	119.8 (3)
C14—C15—C16	120.0 (2)	C35—C36—H36	120.1
C14—C15—H15	120.0	C31—C36—H36	120.1
C16—C15—H15	120.0	C1—N1—H1A	120.0 (17)
C15—C16—C11	119.9 (2)	C1—N1—H1B	119.0 (18)
C15—C16—H16	120.1	H1A—N1—H1B	121 (2)
C11—C16—H16	120.1	C1—N2—N3	120.06 (15)
C22—C21—C26	118.97 (18)	C1—N2—H2	115.7 (18)
C22—C21—P1	123.40 (14)	N3—N2—H2	124.3 (19)
C26—C21—P1	117.55 (14)	N2—N3—H3A	109.2 (19)
C23—C22—C21	120.31 (19)	N2—N3—H3B	106.8 (18)
C23—C22—H22	119.8	H3A—N3—H3B	107 (2)
C21—C22—H22	119.8	C31—P1—C11	103.77 (8)
C24—C23—C22	120.6 (2)	C31—P1—C21	103.33 (8)
C24—C23—H23	119.7	C11—P1—C21	105.00 (8)
C22—C23—H23	119.7	C31—P1—Ag1	117.05 (6)
C23—C24—C25	119.7 (2)	C11—P1—Ag1	109.59 (5)
C23—C24—H24	120.1	C21—P1—Ag1	116.71 (6)
C25—C24—H24	120.1	C1—S1—Ag1	108.17 (5)
C26—C25—C24	120.3 (2)	P1—Ag1—S1	123.445 (15)
C26—C25—H25	119.8	P1—Ag1—Cl1	119.164 (17)
C24—C25—H25	119.8	S1—Ag1—Cl1	111.851 (15)
C25—C26—C21	120.0 (2)	P1—Ag1—Ag1^i^	122.531 (13)
C25—C26—H26	120.0	S1—Ag1—Ag1^i^	58.885 (10)
C21—C26—H26	120.0	Cl1—Ag1—Ag1^i^	105.880 (14)
C32—C31—C36	118.6 (2)		
			
C16—C11—C12—C13	−1.8 (3)	C36—C31—P1—C21	91.93 (16)
P1—C11—C12—C13	178.80 (17)	C32—C31—P1—Ag1	143.54 (15)
C11—C12—C13—C14	0.3 (4)	C36—C31—P1—Ag1	−37.84 (17)
C12—C13—C14—C15	0.7 (4)	C12—C11—P1—C31	−96.91 (17)
C13—C14—C15—C16	−0.2 (3)	C16—C11—P1—C31	83.68 (14)
C14—C15—C16—C11	−1.3 (3)	C12—C11—P1—C21	11.23 (18)
C12—C11—C16—C15	2.3 (3)	C16—C11—P1—C21	−168.18 (13)
P1—C11—C16—C15	−178.27 (14)	C12—C11—P1—Ag1	137.34 (15)
C26—C21—C22—C23	−2.5 (3)	C16—C11—P1—Ag1	−42.07 (14)
P1—C21—C22—C23	−179.27 (18)	C22—C21—P1—C31	18.41 (17)
C21—C22—C23—C24	0.0 (4)	C26—C21—P1—C31	−158.39 (15)
C22—C23—C24—C25	2.0 (4)	C22—C21—P1—C11	−90.05 (16)
C23—C24—C25—C26	−1.3 (5)	C26—C21—P1—C11	93.14 (16)
C24—C25—C26—C21	−1.3 (4)	C22—C21—P1—Ag1	148.38 (14)
C22—C21—C26—C25	3.1 (3)	C26—C21—P1—Ag1	−28.42 (16)
P1—C21—C26—C25	−179.9 (2)	N1—C1—S1—Ag1	−21.93 (16)
C36—C31—C32—C33	−1.7 (3)	N2—C1—S1—Ag1	158.21 (12)
P1—C31—C32—C33	176.89 (16)	C31—P1—Ag1—S1	96.31 (6)
C31—C32—C33—C34	−0.2 (3)	C11—P1—Ag1—S1	−145.95 (6)
C32—C33—C34—C35	1.4 (4)	C21—P1—Ag1—S1	−26.83 (6)
C33—C34—C35—C36	−0.7 (4)	C31—P1—Ag1—Cl1	−55.26 (7)
C34—C35—C36—C31	−1.3 (4)	C11—P1—Ag1—Cl1	62.48 (7)
C32—C31—C36—C35	2.5 (3)	C21—P1—Ag1—Cl1	−178.40 (6)
P1—C31—C36—C35	−176.20 (19)	C31—P1—Ag1—Ag1^i^	168.06 (6)
N1—C1—N2—N3	2.5 (3)	C11—P1—Ag1—Ag1^i^	−74.21 (7)
S1—C1—N2—N3	−177.61 (15)	C21—P1—Ag1—Ag1^i^	44.92 (6)
C32—C31—P1—C11	22.69 (18)	C1—S1—Ag1—P1	−132.43 (5)
C36—C31—P1—C11	−158.69 (16)	C1—S1—Ag1—Cl1	20.96 (6)
C32—C31—P1—C21	−86.69 (17)	C1—S1—Ag1—Ag1^i^	116.84 (5)

Symmetry code: (i) −*x*+2, −*y*+2, −*z*+1.

### Hydrogen-bond geometry (Å, º)

**Table:**

*D*—H···*A*	*D*—H	H···*A*	*D*···*A*	*D*—H···*A*
N3—H3*B*···Cl1^ii^	0.87 (2)	2.67 (2)	3.535 (2)	171 (2)
N2—H2···S1^iii^	0.83 (2)	2.66 (2)	3.4320 (15)	155 (2)
N1—H1*B*···Cl1^iv^	0.85 (2)	2.63 (2)	3.4088 (16)	154 (2)
N1—H1*A*···Cl1	0.89 (2)	2.45 (2)	3.3239 (16)	170 (2)

Symmetry codes: (ii) *x*−1, *y*, *z*; (iii) −*x*+1, −*y*+2, −*z*+1; (iv) −*x*+2, −*y*+3, −*z*+1.
